# Interleukin-2-Inducible T-Cell Kinase (ITK) Deficiency - Clinical and Molecular Aspects

**DOI:** 10.1007/s10875-014-0110-8

**Published:** 2014-10-24

**Authors:** Sujal Ghosh, Kirsten Bienemann, Kaan Boztug, Arndt Borkhardt

**Affiliations:** 1 Department of Pediatric Oncology, Hematology and Clinical Immunology, Medical Faculty, Center of Child and Adolescent Health, Heinrich-Heine-University, Moorenstraße 5, 40225 Duesseldorf, Germany; 2 Institute of Child Health, Molecular and Cellular Immunology Section, UCL Institute of Child Health, 30 Guilford Street, London, WC1N 1EH UK; 3 CeMM Research Center for Molecular Medicine of the Austrian Academy of Sciences, Department of Paediatrics and Adolescent Medicine, Medical University of Vienna, Vienna, 1090 Vienna Austria

**Keywords:** Epstein-Barr virus (EBV), IL-2 inducible kinase (ITK) deficiency

## Abstract

In patients with underlying immunodeficiency, Epstein-Barr virus (EBV) may lead to severe immune dysregulation manifesting as fatal mononucleosis, lymphoma, lymphoproliferative disease (LPD), lymphomatoid granulomatosis, hemophagocytic lymphohistiocytosis (HLH) and dysgammaglobulinemia. Several newly discovered primary immunodeficiencies (*STK4, CD27, MAGT1, CORO1A*) have been described in recent years; our group and collaborators were able to reveal the pathogenicity of mutations in the Interleukin-2-inducible T-cell Kinase (*ITK*) in a cohort of nine patients with most patients presenting with massive EBV B-cell lymphoproliferation. This review summarizes the clinical and immunological findings in these patients. Moreover, we describe the functional consequences of the mutations and draw comparisons with the extensively investigated function of *ITK* in vitro and in the murine model.

## Background

Epstein-Barr virus (EBV), being one of the most common viruses in humans, is best known to cause infectious mononucleosis. In patients with underlying immunodeficiency, EBV may lead to severe immune dysregulation manifesting as fatal mononucleosis, Hodgkin and Non-Hodgkin lymphoma, lymphoproliferative disease (LPD), lymphomatoid granulomatosis, hemophagocytic lymphohistiocytosis (HLH) and dysgammaglobulinemia [1]. Patients, who harbor alterations in genes coding for proteins of the lymphocytic cytotoxic pathway, T cell signaling or T-B cell interaction, may present with EBV associated disease [2]. Mutations in genes of the cytotoxic pathway (e.g. *PRF1)* can lead to hemophagocytic lymphohistiocytosis (HLH) after an infectious trigger, terming these diseases collectively as hemophagocytic syndromes [3, 4]. Furthermore the quite heterogeneous group of combined immunodeficiencies (leaky or atypical SCID) with defects affecting antigen receptor recombination (e.g. hypomorphic mutations in *RAG1/2*) and some distinct genes of T cell signaling (e.g. in *SH2D1A, XIAP*) can lead to EBV associated disease due to immune dysregulation, notably lymphoproliferation [5, 6]. However, EBV pathophysiology varies in each of these diseases.

Several newly discovered primary immunodeficiencies with EBV lymphoproliferation (STK4, CD27, MAGT1, Coronin-1A deficiency) have been described in the last few years [7–11]; our group and collaborators were able to reveal the pathogenicity of mutations in the Interleukin 2-inducible T-cell kinase (*ITK*) gene in a cohort of eight patients all presenting with massive EBV B-cell lymphoproliferation [12–15]. One further patient with recurrent infections and CD4 lymphopenia, but without lymphoproliferation was just recently discovered [16]. Hence, we will briefly summarize the clinical and immunological findings in patients suffering from ITK deficiency and draw comparisons with the extensively investigated function of ITK in vitro and in the murine model.

## Clinical Presentation

In 2009, we described two sisters of consanguineous Turkish descent with EBV lymphoproliferation [12]. The older sister (at the age of 6 years) presented with severe candida stomatitis, pneumocystis jirovici pneumonia, pleural and pericardial effusion, hepatosplenomegaly, cytopenia and progressive hypogammaglobulinemia. A biopsy of an axillary lymph node revealed oligoclonal polymorphic B cell lymphoproliferation of type II latency. Despite repeated courses of Rituximab and partial resolution of clinical symptoms 1 ½ year later the girl developed Hodgkin’s lymphoma. Successfully treated according to standard chemotherapy protocol, T cells unfortunately were continuously declining and the girl died from respiratory failure after acquiring pneumocystis pneumonia at the age of 10 ½ years. The younger sister developed pancytopenia, hepatosplenomegaly, abdominal lymphadenopathy, ascites and pleural effusions due to impaired liver function. EBV associated Hodgkin lymphoma was detected in an inguinal lymph node biopsy; as the clinical situation deteriorated remission could not be achieved and she received haploidentical peripheral blood stem cell transplantation (SCT); unfortunately she died of ischemic brain injury following airway obstruction and cardiac arrest in aplasia. We performed genome-wide linkage analysis with eight family members identifying a region at 5q31-34 that segregated with the disease. Subsequent analysis of candidate genes led to the causative homozygous R335W mutation in the SH2 domain of ITK. Till now we have gathered information on further six patients presenting primarily with massive EBV B-cell lymphoproliferation further progressing to full malignant Hodgkin lymphoma in some cases: three patients were within a family of Palestinian origin [15]; and a total of three single individuals from Iran [14], India and Morocco. Patients with lymphoproliferative disease / Hodgkin lymphoma manifested between 3 and 13 years of age with fever, lymphadenopathy, hepatosplenomegaly and EBV viremia; additional viral infections in some patients included CMV and severe varicella infections indicating a general T cell deficiency. Given severe immune dysregulation, three patients developed autoimmune phenomena (cytopenia, nephritis and thyroiditis); two patients developed HLH. Pulmonary involvement with large interstitial nodules was observed in the majority of patients, as it is known from other EBV-related pathologies; remarkably, extensive pulmonary infiltration of EBV-LPD leading to respiratory distress was the only major primary manifestation in one patient [14]. A ninth ITK deficient patient has just recently been discovered. Interestingly the 18 year-old male Turkish patient of consanguineous background suffered from recurrent progressive pulmonary infections, but no lymphoproliferation so far. Please refer to Table 1 for further details.

**Table 1:** Clinical and laboratory findings in *ITK* deficient patients. abbreviations: AIHA autoimmune hemolytic anemia, Cx chemotherapy, HL Hodgkin Lymphoma, ITP immune thrombocytopenia, LBCL large B-cell lymphoma, LG lymphomatoid granulomatosis, LPD lymphoproliferative disorder, n.d. not determined, n.q. not quantified. ↓ decreased ↘ lower margin ↑ increased

origin/mutation	Patient 1 Turkey c.1003C > T: p.R335W	Patient 2 Turkey c.1003C > T: p.R335W	Patient 3 Palestine c.1764C > G: p.Y588X	Patient 4 Palestine c.1764C > G: p.Y588X	Patient 5 Palestine c.1764C > G: p.Y588X	Patient 6 Morocco c.86G > A: p.R29H	Patient 7 India c.1497delT: p.S499SfsX4	Patient 8 Iran c.468delT: p.P156PfsX109	Patient 9 Turkey c.49C > T: p.Q17X
sex	female	female	female	male	male	male	female	female	male
age at diagnosis	5	6	4	5	3	11	6	13	18
current status	died at age 10	died at age 7	died at age 6	remission after Cx, age 12	well after HSCT, age 8	died at age 26	died after HSCT at age 8	died at age 15	
fever	+	+	+	+	+	+	+	+	+
lymphadenopathy	+	+	+	+	+	+	+	+	none
hepatosplenomegaly	+	+	+	none	+	unknown	none	+	none
pulmonary involvement	+	none	none	+	+	+	+	+	infections
histology	B cell LPD Hodgkin	HL-like B cell LPD	HL	HL	HL	B cell LPD	B cell LPD, LBCL, LG	B cell LPD	none
autoimmunity	none	none	none	nephritis, thyroiditis	thyroiditis	AIHA / ITP	none	none	none
HLH	none	(+)	+ (at relapse)	none	none	none	none	none	none
CD4+ cells	↓	↓	normal	↓	normal	↓	↓		↓
CD8+ cells	normal	↓	↑	↓	normal	normal	normal		normal
NKT cells	n.d.	↘	n.d	↘	n.d.	↘	↘		↘
serology	VCA-G +, VCA-M -, EA-G +, EBNA-G -	VCA-G +, VCA-M–	VCA-G -, VCA-M -, EBNA +	VCA-G +, VCA-M -, EBNA-G -	n.d.	VCA-G +			negative
viral load at presentation	+ (n.q.)	10^3	10^5	10^3	10^5	+ (n.q.)	10^3		10^3
peak viral load	10^7	10^4	unknown	unknown	unknown	10^6	10^4		10^3

## Immune Phenotype, Viral Burden and Lymphoproliferation

Common immunological features in ITK deficient patients are progressive hypogammaglobulinemia and beside global lymphopenia, a progressive loss of CD4+ T cells. Notably, we observe a declining proportion of naive CD45RA+CD4+ T cells. After gating on CD3+ T cells, NKT cells are determined as TCR Vbeta11 and TCR Valpha24 double-positive cells [17]. As seen in other syndromes susceptible to EBV-LPD (e.g. patients with SAP, XIAP, CD27 and Coronin-1A deficiency) NKT cells are severely reduced in the peripheral blood of ITK deficient patients [18]; hence a critical role has been postulated for NKT cells in the response to EBV infection [19]. Patients with EBV-LPD show a rather high EBV viremia though the observed peak viral load in the eight reported patients was quite heterogeneous (10^4 - 10^8 copies/ μg DNA). As the serological phenotype at the time of manifestation differs among the patients, it is only hypothetical to predict the duration between primary infection and clinical immune dysregulation. We did not observe EBV-VCA-IgM in any ITK deficient person; though negative EBNA1-IgG in the first patient could suggest a rather short latency period. No EBV-VCA-IgG seronegative symptomatic EBV-LPD patient has been identified so far (as has been documented in SAP deficiency), leading to the assumption that EBV infection might be essential to cause immune dysregulation in particular LPD, lymphoma and autoimmunity in ITK deficiency. However, the ninth patient lacking EBV-LPD shows a seronegative EBV-VCA status, though revealing a low level of viremia (10^3 copies/ μg DNA). In any case, the early detection and watchful clinical monitoring of ITK deficient patients prior to their first encounter to EBV would be highly desirable.

## Treatment and Outcome

Patients manifested between 3 and 13 years of age. Six out of eight described patients died between 1 and 15 years after primary manifestation of ITK deficiency, five of them within 2 years after the first symptoms appeared. Given the fact, that treatment of EBV-LPD has been best investigated in post-transplant lymphoproliferative disorder (PTLD) patients with a rather type III latency phenotype [20], experience to treat EBV-LPD in primary immunodeficiencies is limited and case-based. Rituximab led to clinical improvement in some ITK deficient patients, while steroids did not show any substantial benefit. However, susceptibility to infections and viremia due to T cell depletion and hypogammaglobulinemia is hardly controlled by antivirals or antibiotics. IgG substitution might be helpful, but however, increasing autoimmunity (nephritis, cytopenia) and Hodgkin lymphoma with the need of chemotherapy and potentially irradiation increase the risk of a fatal outcome. Remarkably, two patients of Palestine origin are alive [15]. While one is in remission after chemotherapy in Hodgkin lymphoma, the other one received a matched sibling bone marrow graft. One girl of Indian origin died after hematopoetic stem cell transplantation due to severe graft-versus-host disease, so at this moment it will remain unclear whether the outcome depends on the nature of disease, pathogenicity of individual mutations or other circumstances unless a bigger cohort is investigated. At least each patient should be evaluated for potential HSCT.

## Interleukin-2-Inducible T-Cell Kinase (ITK)

TEC family kinases comprises five members in mammals namely Bruton agammaglobulinemia tyrosine kinase (BTK), TXK tyrosine kinase (TXK, also RLK), BMX non-receptor tyrosine kinase (BMX, also ETK), tec protein tyrosine kinase (TEC) and Interleukin-2-inducible T-cell kinase (ITK, also LYK) [21]. All proteins function as non-receptor protein-tyrosine kinases in the development and signaling in the lymphoid lineage. X-linked (Bruton’s) agammaglobulinemia, as one of the very first detected primary immunodeficiencies was described in 1952 [22]; the discovery of the corresponding gene happened approximately 40 years later, leading to deep molecular insight into the role of *BTK* in B cell commitment [23]; however studies on ITK deficiency in mice and in vitro were conducted long before the first patient was discovered in 2009. Notably, patients with peripheral T cell lymphoma had been found with translocations of *ITK* with the *SYK* gene and subsequent fusion proteins [24].

The *ITK* gene encompasses a region of 112 kb and 17 exons on chromosome 5q and encodes 620 amino acids of a 71 kDa protein. Like BTK, it consists of a pleckstrin homology (PH) domain at the N-terminus, a Tec homology (TH) domain; a Src homology 3 (SH3) domain; a Src homology 2 (SH2) domain and a catalytic kinase domain at the C-terminus [25]. In the context of adaptive immune response in T and NKT cells antigen presenting cells activate the T cell receptor. Subsequently a series of phosphorylation recruits ITK (which is bound through its pleckstrin homology domain to phosphatidylinositol monophosphates) to the cell membrane, where it is phosphorylated by LCK enhancing autophosphorylation and full activation. ITK itself phosphorylates PLCG1, which subsequent leads to the cleavage of its substrates. Further downstream of this pathway, the endoplasmic reticulum releases calcium in the cytoplasm and the nuclear activator of activated T-cells (NFAT) is translocated into the nucleus for transcriptional activity for further lymphokine production, T cell proliferation and differentiation. Among the six observed pedigrees (nine individuals) two families harbored mutations in the kinase, other three pedigrees had mutations in the SH2 and PH domain. In one patient a deletion led to a truncated SH2 and deleted kinase domain. Interestingly, in *BTK*, homologous mutations have been reported to cause X-linked agammaglobulinemia and the observed mutations in our eight patients have mutations in corresponding *BTK* residues indicating similar structural alterations [13].

Our group did comprehensive studies in transformed Herpesvirus samiri (HVS) cell lines to characterize these mutations and analyze functional consequences. Most mutations did not dramatically change mRNA levels of the *ITK* gene, however taking protein instability into mind, immunoblot analyses of endogenous ITK protein showed several protein variants. Only one patient showed abundance of mRNA levels likely due to nonsense-mediated mRNA decay after a premature stop codon in exon 14 in the kinase region. Protein half-life was determined by pulse-chase studies and showed significant reduced values compared to wild-type ITK. Calcium response was assayed by flux studies in transformed HVS patient T lymphocytes after CD3 antibody dependent TCR stimulation showing clearly reduced or nearly absent cytosolic release of calcium ions in most patients. TCR-mediated calcium mobilization was restored in murine *Itk* −/− thymocytes after transduction of a wild type ITK construct [13].

## *Itk* -/- Murine Phenotype

It is not elaborated how impaired ITK function changes T cell development. Most studies have been undertaken in the *Itk* -/- mouse model with a BALB/c or C57BL/6 background. While, there are even differences in T cell response among these two models it is ambitious to draw a genuine conclusion from mice to human.

In *Itk* -/- mice thymocyte development gives rise to an increased population of innate single positive CD8+ (CD8SP) thymocytes [26]. *Itk* -/- CD8SP thymocytes resemble antigen-experienced T cells showing a CD122 + CD44hiCXCR3+ phenotype with high levels of the transcription factor Eomesodermin (Eomes) and IFNy production upon stimulation. Furthermore splenocytes revealed less CD4 and CD8 expression with a rather mature effector phenotype (CD44 + CD62L+), also showing differential transcriptional signatures with increased levels of Eomes and Tbet as also observed in peripheral CD8 cells of ITK deficient patients [27–31]. As observed in humans, NKT cell development and function is impaired and peripheral survival is reduced [32].

## TH1 Skewing

The current model (in mice and human) of naive CD4 cell differentiation encompasses further maturation to Th1, Th2, Th17, Treg and Tfh cells, not including further more subsets to follow. However, most in vitro and in vivo murine studies addressing the molecular defects of *Itk* −/− CD4 T cells did focus on the Th1/Th2 paradigm giving some evidence that ITK might be responsible for a proper Th2 response [25]. Following TCR stimulation ITK deficient T cells show decreased proliferation and effector cytokine production. In addition to reduced intracellular calcium release, which is shown in human as in mice, an altered NFAT nuclear translocation gives evidence of the biochemical disturbance [33–35]. *Itk* −/− mice have decreased CD4 T cell numbers in the thymus and periphery [26]. Though, ITK seems to be dispensable for general TCR signaling (otherwise the clinical presentation would be more severe), several in-vivo studies of parasite infection in *Itk* −/− mice indicate a role for ITK in Th2 response (see Table 2).

**Table 2:** published in-vivo studies of *Itk* −/− mouse models challenged with parasites

study	Fowell et al., 1999 [33]	Fowell et al., 1999 [33]	Schaeffer et al., 1999 [37]	Schaeffer et al., 2001 [36]
*Itk*−/− background	BALB/c	BALB/c	C57BL6/J	C57BL6/J
parasite	*Leishmania major*	*Nippostrongylus brasiliensis*	*Toxoplasma gondii*	*Schistosoma mansoni*
induced immune response in wt	Th1	Th2	Th1	Th2
effects in *Itk−/−* vs wt	control of infection, IFNy ↑, IL-4 ↓	unable to expel adult worms from the intestines, IL-4 ↓	identical brain cyst count, near normal IFNy production upon ConA and STAg stimulation **BUT** decreased mean survival time	poor granulomatous responses: size of granuloma and draining lymph nodes ↓ᅟIL-4, IL-5 and IL-10 ↑, IFNy ↓

*Itk* −/− mice showed poor granulomatous responses when challenged with *Schistosoma mansoni* eggs, a Th2 response inducing helminth [36]. Beside a marked reduction of size of granuloma and draining lymph nodes, IL-4, IL-5 and IL-10 production was decreased compared to *S. mansoni* infection in the WT. Instead, IFNy production was significantly increased, suggesting ITK deficiency skewed TCR response towards Th1 differentiation. Paradoxically, the same investigations in the *Itk*−/−*Rlk*−/− double knock out mouse (expected to be impaired more severely) did not show any weaker response compared to WT. Another agent to induce a powerful Th2 response is the challenge with the nematode *Nippostrongylos brasiliens* [33]. 12 days after infection with N. brasiliensis wild type BALB/c mice had cleared the worm infection in the gut; in contrast BALB/c mice were unable to expel adult worms and showed a reduction of IL-4 producing cells. Remarkably, *Itk* −/− mice show low susceptibility to some intracellular protozoa [33]. BALB/c mice fail to clear infection with *Leishmania major*, as the usual Th2 response is incapable to clear the parasites; however *Itk* −/− mice show an increase of Th1 dependent IFNy response, leading to the control of infection. The role of *Toxoplasma gondii*, which is usually considered to promote Th1 mediated-immunity, needs to be further elaborated, as *Itk* −/− mice do succumb to this infection [37]. To our knowledge, reports in which *Itk* −/− mice were challenged with the murine herpesvirus 68 (MHV-68), the closest infectious model resembling human EBV infection, have not been published yet. This is somehow surprising given the similarities between MVH-68 and EBV, which were reviewed elsewhere [38–40].

A further approach to examine the Th1/Th2 differentiation was undertaken by several groups investigating the T cell dependent airway hyperresponsiveness in *Itk* −/− mice. Asthma is characterized by the infiltration of Th2 cells into the lungs; *Itk* −/− mice have reduced airway hyperresponsiveness to allergen challenge most likely due to an impaired Th2 response [41]. In humans, only epidemiological studies have focused on SNPs in the *ITK* gene and the susceptibility to developing asthma without any further functional validation [42].

Comprehensive studies beyond the Th1/Th2 paradigm were published by Gomez-Rodriguez 2009 and 2014 [43, 44]. The main focus was the differentiation of Th17 and Treg cells in *Itk* −/− mice. Under Th17 polarizing conditions sorted CD4 cells expressed significantly less IL17A and more FoxP3 RNA compared with wild type cells confirmed by intracellular staining. Moreover, under Treg culture conditions, a larger amount of cells of *Itk* −/− naïve CD4 cells progressed to FoxP3 compared to wild type CD4 cells. Unfortunately, in ITK deficient patients the Th17 and Treg development and function have not been studied yet. The authors further describe a reduced TCR-induced phosphorylation of mammalian target of rapamycin (mTOR) targets accompanied by an altered downstream metabolic profile.

A recent study investigated the protective effect of ITK deficiency on autoimmune phenomena. *CTLA-4* has a quite important function in maintaining T cell tolerance to self, thus *Ctla4 −/−* mice die of an autoimmune lymphoproliferative disorder driven by self-reactive T cell activation and tissue infiltration [45]. In contrast, the *Ctla4−/− Itk*−/− double knockout mouse lacks autoimmune pathology and shows a significant reduced lethality.

## ITK Inhibition

The application of pharmacological ITK inhibitors significantly increased the lifespan of the above mentioned double knockout mouse. The authors further investigated ITK function in the non-obese diabetic mice (NOD), the mouse model of autoimmune insulin-dependent diabetes mellitus, which usually leads to complete destruction of pancreatic beta islets. ITK inhibition by pharmacological means was able to prevent the disease. In contrast to EBV, several viral infections like HIV, Influenza A and Coxsackie virus seem to have a replication cycle depending on ITK [46–48]. Inhibition of ITK leads to inhibition of the replication and alleviation of symptoms in the disease-model. These and many further studies of pharmacological inhibition, often developed from known BTK inhibitors, will portray ITK deficiency from a different perspective. Enthusiastic investigations undertaken to achieve a selective Th1 response is “sought by the medical community given their potential to inhibit a number of Th2-dominant autoimmune, inflammatory, and infectious diseases ranging from cancer immunosuppression and atopic dermatitis to inflammatory bowel disease and even HIV/AIDS” [49, 50]. However to conclude human *ITK* physiology and primary ITK immunodeficiency from these inhibitors is quite ambiguous as this model reflects the acquired rather than the inherited innate ITK impairment.

## Mast Cell Physiology and NK Cell Mediated Cytotoxicity Depends on ITK Function

Given the restricted expression of ITK in T-cells and mast cells, the functional properties of mast cells lacking ITK have been tested in mouse. ITK has been attributed to differentially modulate mast cell degranulation and cytokine production by regulating expression and activation of NFAT proteins. However, the interactions of an allergen-induced mast cell response, serum IgE and histamine release and consecutive altered expression of the FceR are the main features of mast cell dysfunction in the *Itk* −/− mouse [51].

To the best of our knowledge only one study addressed an altered NK function due to ITK impairment. In a human NK cell model, ITK function was inhibited by the application of siRNA; the authors show, that in activated human NK cells, ITK differentially regulates distinct NK-activating receptors. They suggest that ITK positively regulates FcR-initiated cytotoxicity, while in contrast, enhanced ITK expression would negatively regulate NKG2D-initiated granule-mediated killing [52].

## Summary

Over the last 5 years, a couple of patients suffering from inherited ITK deficiency have been reported and it is quite likely that many more have to be identified. After infection with EBV, their clinical symptoms – usually accompanied by ultra-high EBV viral load in the peripheral blood – develop, namely severe lymphoproliferation, and Hodgkin’s lymphoma. The frequent pulmonary involvement seems to emerge as one clinical hallmark, which should alert clinical immunologists to *ITK*. To date, it remains unknown if there is a major pathology in EBV-negative patients as all reported children with LPD were diagnosed after their first encounter with EBV. Definitive treatment recommendations cannot be given yet, but the current knowledge suggests that early stem cell transplantation is life saving. B cell depletion, e.g. by anti-CD20 therapy seem to be only temporary beneficial for those patients.

Both, the physiological role of *ITK* in mouse models and the functional consequences of the loss-of function mutations identified in human patients have been comprehensively studied. While the murine model has been challenged with various parasites to study Th1 and Th2 responses, an “EBV-mimicking” MHV-68 infectious model is warranted to reveal the pathogenesis of EBV infection in ITK deficiency. Furthermore, a more detailed developmental and functional characterization of T-cell subsets (Tregs and Th17 cells) and mast cells lacking ITK will be challenging issues in further studies.
